# Fabrication of an Anti-Reflective and Super-Hydrophobic Structure by Vacuum Ultraviolet Light-Assisted Bonding and Nanoscale Pattern Transfer

**DOI:** 10.3390/mi9040186

**Published:** 2018-04-15

**Authors:** Yuki Hashimoto, Takatoki Yamamoto

**Affiliations:** Department of Mechanical and Control Engineering, Tokyo Institute of Technology, Tokyo 1528552, Japan; hashimoto.y.ai@m.titech.ac.jp

**Keywords:** anti-reflection, super-hydrophobicity, vacuum ultraviolet, polydimethylsiloxane

## Abstract

The application of subwavelength, textured structures to glass surfaces has been shown to reduce reflectivity and also results in self-cleaning due to super-hydrophobicity. However, current methods of producing such textures are typically either expensive or difficult to scale up. Based on prior work by the authors, the present study employed a combination of vacuum ultraviolet (VUV) light-assisted bonding and release agent-free pattern transfer to fabricate a moth-eye texture on a glass substrate. This was accomplished by forming a cyclic olefin polymer mold master with a moth-eye pattern, transferring this pattern to a polydimethylsiloxane (PDMS) spin coating, activating both the PDMS and a glass substrate with VUV light, and then bonding the PDMS to the glass before releasing the mold. Atomic force microscopy demonstrated that the desired pattern was successfully replicated on the PDMS surface with a high degree of accuracy, and the textured glass specimen exhibited approximately 3% higher transmittance than untreated glass. Contact angle measurements also showed that the hydrophobicity of the textured surface was significantly increased. These results confirm that this new technique is a viable means of fabricating optical nanostructures via a simple, inexpensive process.

## 1. Introduction

Glass is one of the most essential components in many optical devices and also has numerous applications in eyeglasses, touch screens, and windows. Glass typically reflects approximately 4% of the light incident on its surface, which both reduces energy efficiency in cases where this light is utilized and generates stray light effects known as flare or ghosting. Thus, suppressing the reflectivity of glass has been an important challenge for about 200 years [1]. Subwavelength structures, such as the so-called moth-eye patterns [2], are known to have a considerable anti-reflective effect over a wide range of incident wavelengths and angles compared with more conventional methods, such as dielectric multilayer coatings [3,4,5]. In addition, these textured surfaces impart a super-hydrophobicity-based self-cleaning feature as a result of the lotus effect [6,7,8]. This phenomenon results in the elimination of dust or pollutant particles from the glass surface via the formation and roll-off of droplets, and is effective at maintaining a clean surface. Recently, an inverted moth-eye structure was also reported to have the same effect, but with greater mechanical robustness, compared to a non-inverted structure [9,10,11,12]. Based on these prior findings, such subwavelength structures have promising applications for both anti-reflection and self-cleaning surfaces. However, the associated fabrication process is relatively complicated, and thus expensive, and so it would be beneficial to develop a low-cost fabrication method to allow the practical use of subwavelength structures. To date, several processes have been proposed, all of which can be roughly divided into three categories. The first type uses lithography and dry etching techniques [13,14,15,16,17]. These approaches have the advantage of being able to generate high-resolution patterns with various shapes, but are relatively costly and have low throughputs, such that it is difficult to apply patterns over large areas. The second type of method is based on the use of self-assembling nanoparticles [5,18,19,20] as dry etching masks. This technique is suitable for large-area patterning, but the nanoparticles themselves are expensive and controlling the shape and arrangement of the nanoparticles is challenging. The third method uses nanoimprinting technologies [21,22,23], which have received much attention with regard to their applications in large-area patterning due to the relatively low cost and high throughput. The nanoimprinting method, however, has some technological drawbacks, including the cost of the imprint mold master and deterioration of the mold pattern due to the use of release agents on the mold [24].

Recently, simple alternative fabrication methods for such periodic structures have been reported, such as electrohydrodynamic inkjet printing method [25] and electrohydrodynamic assembly method [26]. In a previous study, we developed and demonstrated a simple, facile patterning method by taking advantage of a combination of vacuum ultraviolet (VUV) bonding and the release agent-free molding of polydimethylsiloxane (PDMS) using a cyclic olefin polymer (COP) mold master [27]. This technique has several advantages compared to conventional methods. Specifically, the COP mold master can be duplicated from a template mold and does not require any release agent to transfer PDMS-made nanostructures, due to its low surface energy [28]. As a result, the fabrication process is simplified, and is also made more accurate by eliminating the release agent layer from the mold master surface. This approach also simplifies the bonding between PDMS-made nanostructures and Si-based materials such as Si, glass, and plastics, which can be accomplished simply by irradiation with VUV light. PDMS also exhibits comparable or improved performance relative to conventional optical polymers in terms of optical transparency [29] and resistance to chemical corrosion [30,31]. Furthermore, the COP-made mold master can itself be replicated from the original template, which reduces the cost of fabricating the mold master for PDMS replication. Therefore, this method should be well suited to the production of subwavelength structures at low cost and with high throughput.

Based on the above, in the present work, we developed and demonstrated a new technique for making inverted moth-eye structures on a glass substrate, and evaluated the effectiveness of the resulting structures as antireflection and super-hydrophobic surfaces.

## 2. Materials and Methods

### 2.1. Materials and Design

UV-curable PDMS (UV-PDMS) (X-34-4184) was selected as the material on which the inverted moth-eye structure would be generated because of the unique characteristics of this polymer. These include ready release from the mold, high transparency in the visible light region, chemical resistivity, and extremely low thermal shrinkage during curing compared with conventional thermosets polymer [27,32,33].

The pitch of the inverted moth-eye structure (*p*) was determined based on Equation (1), which defines the pitch range over which a structure is anti-reflective in response to normally incident light [14].

(1) $$p<\frac{\lambda }{n}$$

Here, *p* is assumed to be the same as the width of the moth-eye structure, *n* is the refractive index of the material used to produce the moth-eye structure and *λ* is the incident wavelength. To suppress the reflection of normally incident light at visible wavelengths (ranging from 400 to 700 nm), *n* and *λ* values of 1.43 [10] and 400 nm were employed, respectively. These values give a pitch of 250 nm and thus satisfy the criterion in Equation (1).

**Figure 1:**
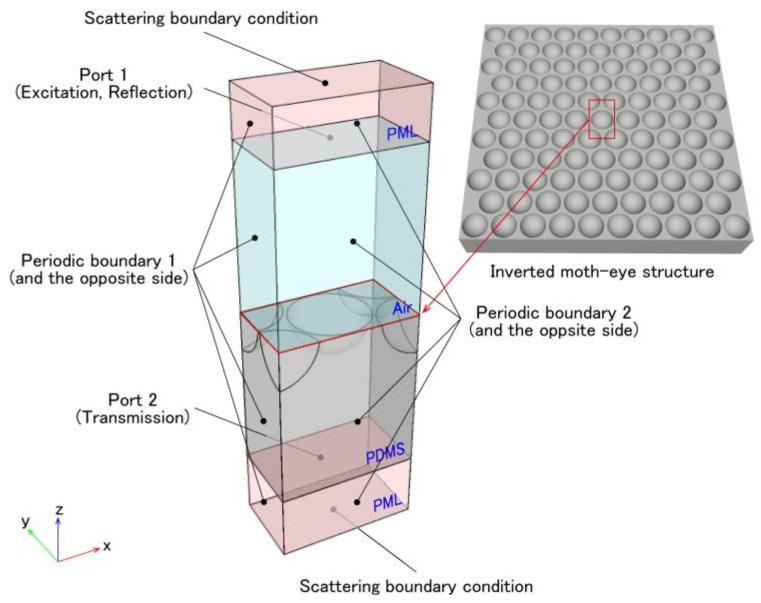
Unit cell employed as a finite element model of the inverted moth-eye structure in the COMSOL Multiphysics RF module.

The optimum depth of the inverted moth-eye structure was determined by finite element analysis using a commercially available software package (COMSOL Multiphysics, COMSOL Inc., Stockholm, Sweden). The periodic nature of this structure meant that a simulation could be performed based on a unit cell with appropriate boundary conditions. The radio frequency (RF) module in COMSOL allows an incident plane wave of visible light with a given intensity and propagation direction to penetrate a surface in conjunction with scattering, and Figure 1 shows the configuration of the unit cell used in the simulations. The electromagnetic fields in unbounded models were calculated using perfectly matched layers (PMLs) on the edges of the models to absorb the outgoing electromagnetic waves [34]. Perfect magnetic and electric conductors were employed as periodic boundary conditions for normal incidence at periodic boundaries 1 and 2, respectively [35,36]. The combination of active port 1 and passive port 2 allowed the use of a scattering parameter for the structure (*S*) to determine both the reflection and transmission [34]. In this simulation, the inverted moth-eye structure was assumed to have an ellipsoidal cross-section, and the pitch and width were fixed at 250 nm based on Equation (1). 

**Figure 2:**
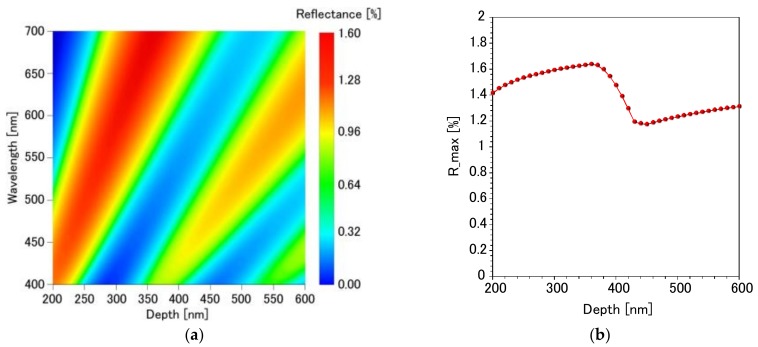
(**a**) Simulated reflection spectrum of the inverted moth-eye structure at various depths (200–600 nm) and incident light wavelengths (400–700 nm) The color bar indicates the reflectance values. (**b**) Dependence of the calculated maximum reflectance (R_max) in the visible light region on the depth of the inverted moth-eye structure.

Figure 2a presents the simulated reflection spectrum of a PDMS specimen having an inverted moth-eye structure as a function of depth. It is evident that the moth-eye pattern suppresses reflection on the substrate compared to the reflectance value of 3.1% from a flat surface. Figure 2b plots the maximum reflectance in the visible light region as a function of the depth of the inverted moth-eye structure. A minimum is seen to occur at a depth of 450 nm, and so this value was adopted for subsequent experimental trials.

### 2.2. Fabrication

**Figure 3:**
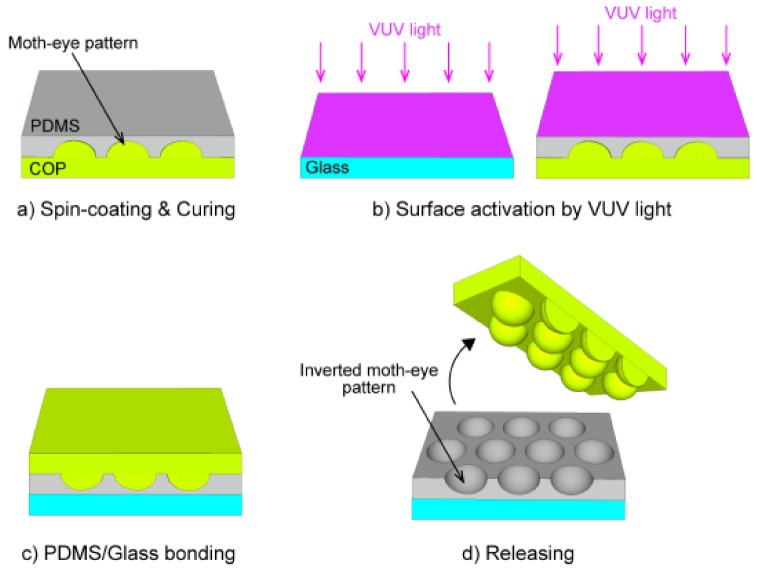
Schematic diagram of the VUV-assisted bonding and nanoscale pattern transfer process for fabricating the inverted moth-eye structure. (**a**) Spin-coating and curing of PDMS on a COP mold with a textured pattern, (**b**) surface activation of the glass and PDMS by VUV light, (**c**) bonding of the glass and the PDMS surfaces, and (**d**) release of the COP mold from the PDMS layer.

Figure 3 presents a diagram summarizing the VUV-assisted bonding and nanoscale pattern transfer process used to impart an inverted moth-eye structure to PDMS on a glass substrate [27]. The UV-PDMS is first spin-coated onto a COP master mold with a textured pattern and then cured by irradiation with UV light (Figure 3a), following which both the PDMS and the glass substrate surfaces are activated by exposure to VUV irradiation (Figure 3b) and then bonded to one another (Figure 3c). Finally, the master mold is released from the PDMS layer to obtain an inverted moth-eye morphology (Figure 3d).

This structure was obtained by first fabricating a COP master mold by thermal nanoimprinting using a silicon template on which a nanoscale pattern had been produced. This silicon etching mask was obtained using electron beam (EB) lithography and dry etching to impart the inverted moth-eye structure to the silicon, followed by removal of the EB resist by oxygen plasma ashing. In the initial step, an EB apparatus (F7000S-VD02, Advantest Co., Ltd., Tokyo, Japan) was employed to fabricate a nanoscale array of holes (50 nm in diameter) on a positive-type EB resist (ZEP-520A, Zeon Co., Ltd., Tokyo, Japan). Subsequently, the inverted moth-eye pattern was fabricated on the silicon substrate by a dry etching (ASE-SR/SRE, SPP Technology Co., Ltd., Tokyo, Japan), using SF_6_ as the etching gas at a flow rate of 13.0 mL/min and a pressure of 2.3 Pa, in conjunction with an RF power of 100 W and etching time of 300 s. During the ashing process, we employed an oxygen plasma ashing apparatus (PR510, Yamato Scientific Co., Ltd., Tokyo, Japan) with an oxygen flow rate of 200 mL/min and an RF power of 500 W, applied for 60 s.

The thermal nanoimprinting was performed by applying a pressure of 6 MPa to the COP film (ZF-14, ZEON Co., Ltd., Tokyo, Japan) so as to transfer the nanoscale pattern onto the silicon template, while heating at 170 °C for 300 s. After cooling to 120 °C, the COP film was detached from the silicon template. The UV-PDMS pre-polymer was poured onto the mold and spin-coating was performed at 4000 rpm for 60 s. The UV-PDMS was then cured by exposing it to UV light at 365 nm, with an irradiation dose of 2000 mJ/cm^2^ at room temperature. The surfaces of the PDMS and glass were then irradiated with VUV light with an irradiation dose of 125 mJ/cm^2^ using a water-cooled dielectric barrier discharge excimer lamp filled with xenon gas (UVS-1000SM, Ushio Co., Ltd., Tokyo, Japan) that emitted incoherent light at a wavelength of 172 nm [27]. This irradiation was carried out in air with a distance of 3 mm between the lamp surface and the specimen. Following this, the glass substrate and PDMS surface were brought into contact with one another without any pressure load. After 30 min, the master mold was detached, leaving the PDMS structure on the glass substrate.

After fabrication, we used atomic force microscopy (AFM; Dimension Icon, Bruker Co., Ltd., Billerica, MA, USA) to evaluate the shape of the COP mold and that of the inverted moth-eye structure made of PDMS.

### 2.3. Optical Measurements

The optical properties of the inverted moth-eye structure were evaluated by measuring visible light transmittance with a spectrophotometer (VT-770, JASCO Co. Ltd., Tokyo, Japan) and comparing the experimental data with the simulated results. The total transmittance, *T_total_*, through the PDMS (having an inverted moth-eye structure) and the glass substrate was estimated using Equation (2). This calculation ignored visible light absorption in the PDMS and glass and secondary or greater reflections because these values were relatively low.

(2) $$T_{total}\approx \left(1-R_{Air/PDMS}\right)\left(1-R_{PDMS/Glass}\right)\left(1-R_{Glass/Air}\right)$$

Here, *R_Air/PDMS_*, *R_PDMS/Glass_*, and *R_Glass/Air_* are the reflectance of the PDMS inverted moth-eye structure, the PDMS/glass interface, and the glass/air interface, respectively. *R_Air/PDMS_* was obtained from the simulation results in Figure 2, and *R_PDMS/Glass_* was calculated using Equation (3), below.

(3) $$R_{PDMS/Glass}={\left(\frac{n_{PDMS}-n_{Glass}}{n_{PDMS}+n_{Glass}}\right)}^{2}$$

Here, *n_PDMS_* and *n_Glass_* are the refractive index of the PDMS and glass, respectively. The value of *n_PDMS_* was fixed at 1.43 [18] and *n_Glass_* was calculated using Equation (4).

(4) $$n_{Glass}=\frac{1-\sqrt{R_{Glass/Air}}}{1+\sqrt{R_{Glass/Air}}}$$

*R_Glass/Air_* was calculated using Equation (5).

(5) $$R_{Glass/Air}=1-\sqrt{T_{Air/Glass/Air}}$$

### 2.4. Contact Angle Measurements

The contact angle of water on the sample surface was determined to evaluate the surface hydrophobicity [37]. This measurement was performed using a CCD camera (MC681SPD, Texas Instruments Co. Ltd., Dallas, TX, USA) to capture a side-view of a 15 µL water droplet on the sample surface and the result was compared with the theoretical value based on the Gibbs free energy (GFE) approach. The GFE stands for the free energy of the whole system containing air, the droplet, and the substrate. The GFE density (G*) at the solid-liquid interface on the substrate was calculated using Equation (6) [38].

(6) $$G^{*}=\frac{\left\{2-2cos\theta_{E}^{*}-sin^{2}\theta_{E}^{*}\left(r_{\phi }\phi_{s}\cos\theta +\phi_{s}-1\right)\right\}}{4^{\frac{1}{3}}{\left(2-3cos\theta_{E}^{*}+\cos^{3}\theta_{E}^{*}\right)}^{\frac{2}{3}}}$$

**Figure 4:**
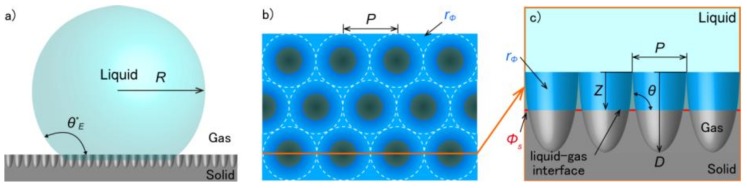
Schematic diagram of a liquid droplet on a textured surface similar to the inverted moth-eye structure, showing the parameters used to calculate the GFE density: (**a**) side-view, (**b**) enlarged top-view, and (**c**) enlarged side-view corresponding to the line in the top-view. Areas in deep blue indicate the ratio of the actual wetted area to the total solid area of the textured surface ($r_{\phi }$), and red areas indicate the ratio of the actual area of liquid-solid contact to the projected area on the horizontal plane ($\phi_{s}$).

Here, G* is the GFE density, $\theta_{E}^{*}$ is the estimated apparent contact angle, θ is the contact angle on a flat substrate, $\phi_{s}$ is the ratio of the actual area of liquid-solid contact to the projected area on the horizontal plane, and $r_{\phi }$ is the ratio of the actual wetted area to the total solid area of the textured surface shown in Figure 4. The terms $\phi_{s}$ and $r_{\phi }$ were calculated as functions of the normalized vertical position (*Z*/*D*) and the period of the structure (*P*) using Equations (7) and (8), respectively.

(7) $$\phi_{s}=\frac{P^{2}}{8}\left[2\sqrt{3}-\pi \left\{1-{\left(\frac{Z}{D}\right)}^{2}\right\}\right]$$

(8) $$r_{\phi }=1-\frac{\pi }{2\sqrt{3}}+\frac{\pi D}{\sqrt{3}P}\left\{\sin^{-1}\left(\frac{Z}{D}\right)+\frac{Z}{D}\sqrt{1-{\left(\frac{Z}{D}\right)}^{2}}\right\}$$

To compute the GFE density (G*) and determine the apparent contact angle ($\theta_{}^{*}$) and the wetting state corresponding to the global energy minimum (min(G*)), a code was developed using the MATLAB software package, wherein the values of $\theta_{E}^{*}$ and *Z*/*D* were simultaneously varied from 0° and 180° and from 0 to 1, respectively, in steps of 0.5° and 0.01.

## 3. Results and Discussion

### 3.1. Fabrication

**Figure 5:**
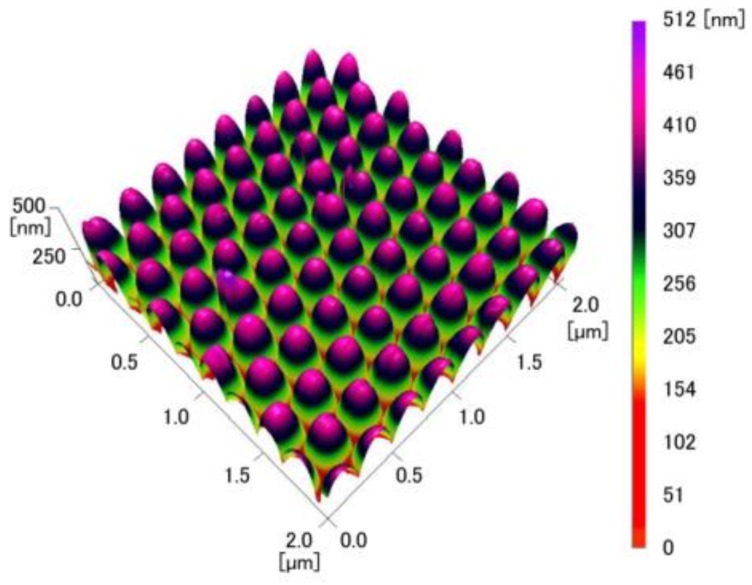
AFM image of the COP mold.

**Figure 6:**
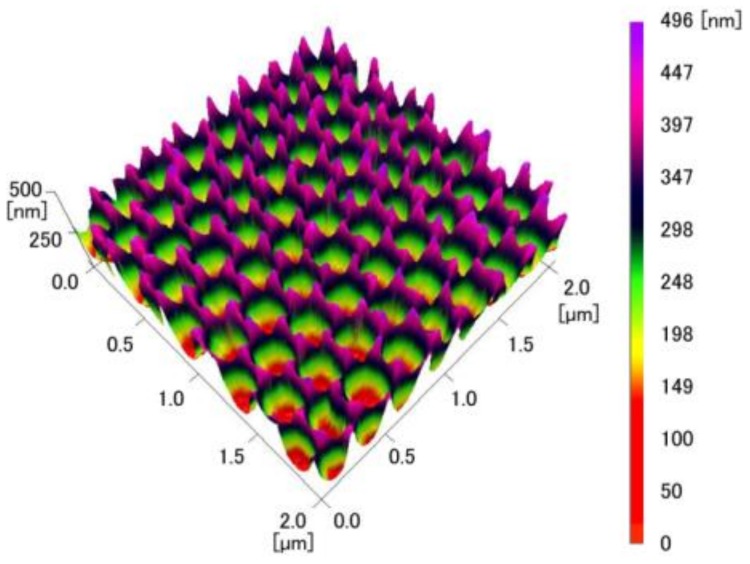
AFM image of the inverted moth-eye structure made of PDMS.

The AFM images of the COP mold and the inverted moth-eye structure made of PDMS in Figure 5 and Figure 6 confirm that the COP mold had a regular moth-eye structure with an average period and height of 249.8 nm and 449.8 nm, respectively. The final structure in the PDMS had an inverted moth-eye pattern almost identical to the pattern on the silicon master mold, with an average period and depth of 249.7 nm and 449.5 nm, respectively. In general, AFM or SEM measurement usually has errors of only a few percent. Thus, these data demonstrate that an inverted moth-eye structure almost the same size as the pattern in the original silicon master mold was successfully fabricated, with the pitch and the depth within 10 nm of the nominal values.

### 3.2. Optical Measurements

**Figure 7:**
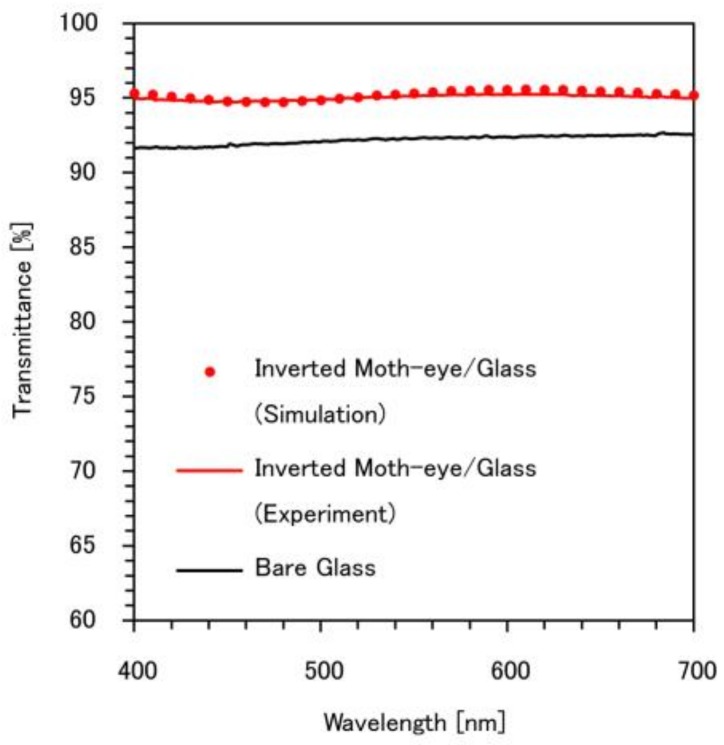
Transmission spectrum of a glass substrate bonded to PDMS having an inverted moth-eye structure. The spectrum for glass and the simulated spectrum are shown for comparison purposes.

Figure 7 provides the transmission spectrum of the glass substrate with the PDMS inverted moth-eye structure fabricated as described above. According to these results, the experimental transmittance of the PDMS inverted moth-eye structure on the glass was approximately 3% higher than the transmittance of the glass alone. This is because the presence of the inverted moth-eye structure decreases reflectance compared with the bare flat glass. In the case of the bare glass, about 4% of incident light was reflected on the surface, whereas the reflectance was achieved less than 1% in the case of the surface with the PDMS inverted moth-eye structure. Therefore, the transmittance was increased after fabricating the inverted moth-eye structure on the bare glass. In addition, the simulation results are in good agreement with the experimental data, with the experimental values being slightly lower than the simulation. This discrepancy may be the result of minor variations in the unit cell dimensions in the fabricated structure.

### 3.3. Contact Angle Measurements

**Figure 8:**
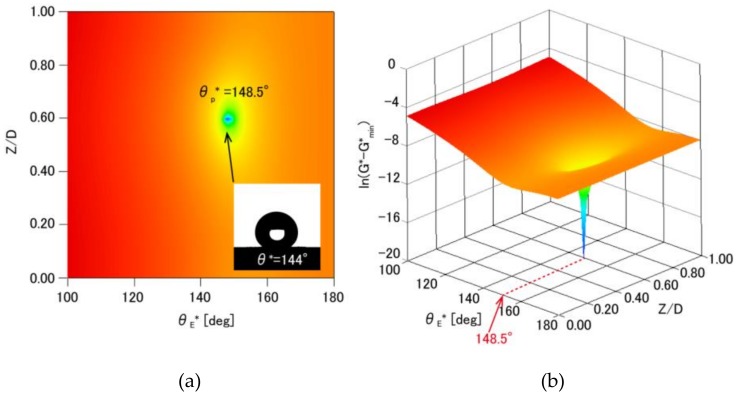
Contour maps (2D map:(a), 3D map:(b)) showing changes in the GFE density (In(G* − G*_min_)) as a function of the estimated apparent contact angle ($\theta_{E}^{*}$) with the normalized position at the water-air interface (*Z*/*D*) in an inverted moth-eye structure made of PDMS (θ=100°). The insert in the 2D map is a captured image of a water droplet on the inverted moth-eye structure. The blue region represents the global minimum in the GFE density.

Figure 8 summarizes the water contact angles on an inverted moth-eye structure made of PDMS by plotting variations in the GFE density (In(G* − G*_min_)) as a function of the estimated apparent contact angle ($\theta_{E}^{*}$). These results show that a larger contact angle was obtained with the moth-eye structure compared with that resulting from a flat surface (100°). The GFE density is minimized at *Z*/*D* = 0.60 and $\theta_{E}^{*}$ = 148.5°, as indicated by the blue regions in these maps. This angle is in good agreement with the experimental value of 144°. Through this comparison, this calculation method is usable to estimate wettability of the substrate with these periodic structures.

## 4. Conclusions

In this work, we employed a simple, facile VUV light-assisted bonding and nanoscale pattern transfer technique developed and demonstrated in a previous study to fabricate an inverted moth-eye structure. We succeeded in producing the desired structure over the top of a glass substrate and the resulting unit exhibited high transparency and super-hydrophobicity through the optical and contact angle measurement. It is apparent that this method can be applied to the construction of subwavelength structures such as an inverted moth-eye structure.

In addition, Fabrication using a deformable COP mold master, as demonstrated herein, also has the potential to produce nanostructures on curved surfaces. Hence, in the future, one application of this technique could be the fabrication of optical nanostructures on curved surfaces, such as anti-reflection moth-eye structures on optical lenses, glasses, curved display panels, and curved solar cells. Such structures would improve the transmittance or absorption of light and produce self-cleaning surfaces, both of which are difficult to realize by conventional nanoimprinting using hard mold masters.
